# Salinomycin inhibits prostate cancer growth and migration via induction of oxidative stress

**DOI:** 10.1038/bjc.2011.530

**Published:** 2012-01-03

**Authors:** K Ketola, M Hilvo, T Hyötyläinen, A Vuoristo, A-L Ruskeepää, M Orešič, O Kallioniemi, K Iljin

**Affiliations:** 1 Medical Biotechnology, VTT Technical Research Centre of Finland, University of Turku, PL 106, FI-20521 Turku, Finland; 2 VTT Technical Research Centre of Finland, FI-02044 VTT, Finland; 3 Institute for Molecular Medicine, Finland (FIMM), University of Helsinki, FI-00014 Helsinki, Finland

**Keywords:** salinomycin, prostate cancer, oxidative stress, cancer stem cells

## Abstract

**Background:**

We have shown that a sodium ionophore monensin inhibits prostate cancer cell growth. A structurally related compound to monensin, salinomycin, was recently identified as a putative cancer stem cell inhibitor.

**Methods:**

The growth inhibitory potential of salinomycin was studied in a panel of prostate cells. To get insights into the mechanism of action, a variety of assays such as gene expression and steroid profiling were performed in salinomycin-exposed prostate cancer cells.

**Results:**

Salinomycin inhibited the growth of prostate cancer cells, but did not affect non-malignant prostate epithelial cells. Salinomycin impacted on prostate cancer stem cell functions as evidenced by reduced aldehyde dehydrogenase activity and the fraction of CD44^+^ cells. Moreover, salinomycin reduced the expression of *MYC*, *AR* and *ERG,* induced oxidative stress as well as inhibited nuclear factor-*κ*B activity and cell migration. Furthermore, profiling steroid metabolites revealed increased levels of oxidative stress-inducing steroids 7-ketocholesterol and aldosterone and decreased levels of antioxidative steroids progesterone and pregnenolone in salinomycin-exposed prostate cancer cells.

**Conclusion:**

Our results indicate that salinomycin inhibits prostate cancer cell growth and migration by reducing the expression of key prostate cancer oncogenes, inducing oxidative stress, decreasing the antioxidative capacity and cancer stem cell fraction.

Redox regulation has an important role in controlling cancer cell behaviour. Cancer cells may potentially benefit from oxidative stress induction and production of reactive oxygen species (ROS), which are known to increase the rate of mutations (Auten and Davis, 2009; Acharya *et al*, 2010; Cairns *et al*, 2011). However, as cancer cells have a higher level of oxidative stress than non-malignant cells, cancer cells are dependent on an active antioxidant defence system. Thus, cancer cells are vulnerable to acute induction of oxidative stress caused by agents inducing ROS or reducing antioxidative capacity (Khandrika *et al*, 2009; Trachootham *et al*, 2009; Acharya *et al*, 2010; Cairns *et al*, 2011).

Interestingly, most key genes involved in prostate cancer modulate the antioxidative capacity. For example, the decrease in androgen receptor (AR) signalling has been shown to reduce the antioxidative capacity and increase ROS production (Tam *et al*, 2003; Pinthus *et al*, 2007), MYC expression is known to protect cells against oxidative stress (Benassi *et al*, 2006) and our recent results indicate that transmembrane protease, serine 2 (TMPRSS2)–the v-ets erythroblastosis virus E26 oncogene homolog (ERG) fusion-positive VCaP prostate cancer cells are vulnerable to oxidative stress induction (Iljin *et al*, 2009; Ketola *et al*, 2010; Vainio *et al*, 2011). Moreover, recent studies have shown that the enzymes that are used as cancer stem cell and tumour-initiating cell markers have antioxidative properties. Aldehyde dehydrogenase (ALDH) activity, used as a marker for prostate cancer stem cells (Moreb, 2008; Burger *et al*, 2009; Li *et al*, 2010; Yu *et al*, 2011), has an important role in maintaining antioxidative capacity and CD44, known to characterise cancer stem cells and to correlate with the ability of prostate cancer cells to migrate, regulates redox status in cancer cells (Klarmann *et al*, 2009; Ishimoto *et al*, 2011). Thus, these studies indicate that modulation of the redox status could be a potential therapeutic avenue to impact on prostate cancer cells, including the cancer-initiating cells.

We have recently identified monensin as a novel oxidative stress inducer and antineoplastic compound in prostate cancer cells (Iljin *et al*, 2009; Ketola *et al*, 2010). Interestingly, salinomycin, a structurally similar ionophorous antibiotic as monensin, was recently identified as a breast cancer stem cell inhibitor *in vitro* and shown to inhibit breast cancer xenograft growth *in vivo* (Gupta *et al*, 2009). Salinomycin reduces cancer and cancer stem cell growth also in other tumour types, such as leukaemias and uterine sarcoma cells (Fuchs *et al*, 2009, 2010). Traditionally, salinomycin has been used as an antimicrobial agent in veterinary medicine (Miyazaki *et al*, 1974; Mahmoudi *et al*, 2006) and it has been shown to inhibit oxidative phosphorylation in mitochondria by increasing the cation transport across the mitochondrial membrane (Mitani *et al*, 1976). Results from recent mechanistic studies in breast cancer cells indicate that salinomycin can induce DNA damage (Kim *et al*, 2010). However, the effect of salinomycin exposure on prostate cancer growth has not been studied before. Therefore, in this study, we explored the growth inhibitory potential of salinomycin in cultured prostate cancer and non-malignant prostate epithelial cells and studied its mechanism of action focusing on the relationship between antioxidant and cancer-initiating cell properties.

## Materials and Methods

### Cells

VCaP prostate carcinoma cells (*TMPRSS2–ERG* positive, received from Drs Adrie van Bokhoven, University of Colorado Health Sciences Center, Denver, CO, USA and Kenneth Pienta, University of Michigan, Ann Arbor, MI, USA) were grown in Dulbecco's modified Eagle's medium (Korenchuk *et al*, 2001). LNCaP prostate carcinoma cells (received from Dr Marco Cecchini, University of Bern, Bern, Switzerland) were grown in T-Medium (Invitrogen Molecular Probes, Carlsbad, CA, USA). The non-malignant RWPE-1 prostate epithelial cells (Webber *et al*, 1997) and prostate carcinoma cells PC-3 and DU 145 were purchased from American Type Culture Collection (LGC Promochem AB, Borås, Sweden) and grown according to provider's instructions. The non-malignant EP156T prostate epithelial cells were received from Dr Varda Rotter (Weizmann Institute of Science, Rehovot, Israel) and grown in the media recommended by the distributor (Kogan *et al*, 2006). Primary prostate epithelial cells were ordered from Lonza Walkersville, Inc. (Walkersville, MD, USA).

### Compounds

Salinomycin and vitamin C were purchased from Sigma-Aldrich (St Louis, MO, USA) and diluted in DMSO.

### Cell viability and apoptosis assays

Cell viability and apoptosis assays were done on Falcon 384-well plates (BD Biosciences, San Jose, CA, USA) as previously described (Ketola *et al*, 2010). Cell viability was determined with CellTiter-Glo cell viability assay (Promega, Madison, WI, USA) and induction of caspase 3 and 7 activities was detected with homogenous Apo-ONE assay (Promega) according to the manufacturer's instructions. The signals were quantified using Envision Multilabel Plate Reader (Perkin-Elmer, Massachusetts, MA, USA).

### Determination of ALDH activity

The activity of ALDH in response to 1 *μ*M salinomycin exposure in prostate cancer cells was determined with Aldefluor reagent (Stemcell Technologies, Vancouver, BC, Canada) as previously described (Ketola *et al*, 2010). ALDH inhibitor diethylaminobenzaldehyde (DEAB) was used as a negative control. The fluorometric signal was determined with Envision Multilabel Reader (Perkin-Elmer).

### Fluorescence-activated cell-sorting analysis

VCaP, LNCaP, PC-3 and DU-145 cells were exposed to salinomycin (1 *μ*M) for 6 h, samples were fixed with 2% paraformaldehyde and stained with CD44 (FITC-conjugated mouse monoclonal anti-human, BD Pharmingen (San Diego, CA, USA) 555478, BD Pharmingen) antibody for 45 min at 4°C in the dark. Cells were washed and the fluorescence intensity was measured using Accuri C6 Flow Cytometer (BD Accuri Cytometers, Ann Arbor, MI, USA).

### Gene expression analysis using bead arrays

VCaP cells were treated with 1 *μ*M salinomycin for 3, 6 and 24 h, total RNA was extracted and RNA integrity was monitored using an Experion electrophoresis station (Bio-Rad Laboratories, Hercules, CA, USA). Purified RNA (300 ng) was used for amplification with the Illumina RNA TotalPrep Amplification kit (Ambion, Austin, TX, USA) and the biotin-labeled cRNA was hybridised to Sentrix HumanRef-8 *vs* 3 Expression BeadChips (Illumina, San Diego, CA, USA). The arrays were scanned with the BeadArray Reader (Illumina).

### Statistical analysis of gene expression data

The raw gene expression data were quantile-normalised (Gentleman *et al*, 2004) and analysed as previously described (Ketola *et al*, 2010). The gene names from Illumina experiments were rendered to Affymetrix gene IDs with Ensemble Genes 59 database. Ingenuity Pathway Analysis (IPA) software (Ingenuity Systems Inc., Redwood City, CA, USA) was used to analyse the functional gene ontology and pathway annotations. Differentially expressed genes (logFC >0.5 or <−0.5) were selected for the IPA analysis. Connectivity Map 02 was used to identify drugs with similar or opposite effects on gene expression (Lamb, 2007).

### Statistical analyses

The error bars in the figures are shown as standard deviations. The asterisks indicate statistical significance. ^*^P<0.05; ^**^P<0.01; ^***^P<0.001.

### RNA extraction and quantitative reverse transcriptase PCR

Total RNA was extracted and quantitative real-time PCR was done as previously described (Ketola *et al*, 2010). TaqMan gene expression probes and primers from the Universal Probe Library (Roche Diagnostics, Espoo, Finland) were used to study AR, prostate-specific antigen (PSA), ERG, MYC, Kruppel-like factor 6 (KLF6) and activating transcription factor 3 (ATF3), metallothioneins MT1G and MT1F, thioredoxin-binding protein (TXNIP), DNA damage-inducible transcripts 3 and 4 (DDIT3 and DDIT4) and *β*-actin mRNA expression (Supplementary Table 1). Three replicate samples were studied for quantitation of mRNA expression.

### Western blot analysis

Western blot analysis was performed for compound-treated samples using specific antibodies against AR (1 : 1000 dilution, mouse monoclonal, Labvision, Fremont, CA, USA), PSA (1 : 1000 dilution, rabbit polyclonal, DakoCytomation, Glostrup, Denmark) and *β*-actin (1 : 4000 dilution, mouse-monoclonal, Becton Dickinson, Franklin Lakes, NJ, USA). Signal was detected with 1 : 4000 dilution of appropriate HRP-conjugated secondary antibodies (all from Invitrogen Molecular Probes, Carlsbad, CA, USA) followed by visualisation with the enhanced chemiluminescence reagent (Amersham Biosciences, Little Chalfont, UK).

### Reactive oxygen species detection

The intracellular ROS was measured in response to salinomycin exposure for 48 h with carboxy-H2DCFDA as previously described (Ketola *et al*, 2010). As a positive ROS control, hydrogen peroxide exposure (400 *μ*M) for 4 h was used. The oxidation of the probe was measured in PBS by monitoring the increase in fluorescence with Envision Multilabel Plate Reader (Perkin Elmer).

### Cancer luciferase reporter assay

The activity of nuclear factor-*κ*B (NF-*κ*B) signalling pathway was measured using luciferase reporter assay (SABiosciences, Frederick, MD, USA). In brief, inducible NF-*κ*B transcription factor responsive firefly luciferase reporter with constitutively expressing Renilla construct transcription factor reporter were transfected in prostate cancer cells. A mixture of non-inducible firefly luciferase reporter and constitutively expressing Renilla construct was used as a negative control. After 24 h, salinomycin (100 nM) or control were added onto the cells for 18 h. The Dual-LuciferaseReporter (DLR) Assay System (Promega) was used to measure the luciferase activities and results were analysed according to the manufacturer's instructions.

### Wound-healing assay

The effect of salinomycin (100 nM and 1 *μ*M) alone and in combination with vitamin C (10 *μ*M) on prostate cancer cell migration was studied using a wound-healing assay. PC-3 cells were plated on 96-well plates (Essen ImageLock, Essen Instruments, Birmingham, UK) and a wound was scratched with wound scratcher (Essen Instruments). Compounds and appropriate controls were added immediately after wound scratching and wound confluence was monitored with Incucyte Live-Cell Imaging System and software (Essen Instruments). Wound closure was observed every hour for 24 h by comparing the mean relative wound density of three biological replicates in each experiment.

### Steroid quantification

VCaP cells were exposed to 1 *μ*M salinomycin for 6 h, harvested and counted. An internal standard (labelled C16 : 0) and chloroform/methanol (2 : 5) mixture were added, the samples were homogenised with Retsch system (5 min, 20 Hz), centrifuged and the supernatant was collected and evaporated. MOX (25 *μ*l, TS-45950, Thermo Scientific, Helsinki, Finland) was added and the mixture was incubated at 45°C for 60 min. Next, 100 *μ*l of MSTFA with 1% trimethylchlorosilane (Fluka, St Louis, MO, USA) was added and the mixture was incubated at 70°C for 60 min. Injection standard was added to the mixture before gas chromatography-mass spectrometry analysis (GC-MS, Agilent 6890 gas chromatograph (GC) combined with Agilent 5973 mass selective detector (MSD), Agilent Technologies, Espoo, Finland). The injector (injection volume 1 *μ*l with pulsed splitless injection) and MSD temperatures were 230°C (MS Source) and 150°C (MS Quad). The analyses were performed on Supelco 38499-02C capillary column. Selective ion monitoring using specific masses for each target analyte was used in the detection. The following steroids were quantified: 7-ketocholesterol, aldosterone, progesterone, pregnenolone, estrone, 17B-estradiol, 4B-hydroxycholesterol, 25-hydroxycholesterol, 5a,6a-epoxycholesterol (Mono-TMS), dihydrotestosterone and testosterone (the standards were from Steraloids, Newport, RI, USA).

## Results

### Salinomycin inhibits prostate cancer cell growth but does not induce apoptosis

The effect of salinomycin on cell growth was studied in panel of malignant (VCaP, LNCaP, PC-3, DU 145) and non-malignant (RWPE-1, EP156T and PrEC) prostate cells. Interestingly, salinomycin was the most effective in inhibiting VCaP cells (EC_50_=380 nM), whereas non-malignant prostate epithelial cells RWPE-1, EP156T and PrEC were non-responsive (EC_50_>10 *μ*M) (Table 1). Salinomycin was also at least 10-fold more potent growth inhibitor in other prostate cancer cells studied compared with non-malignant prostate epithelial cells (Table 1). To determine whether salinomycin induces apoptosis in VCaP and LNCaP cells, caspase 3 and 7 activities were determined by a quantitative fluorometric assay. No significant increase in caspase activity was observed in response to salinomycin exposure (up to 10  *μ*M) for 48 h in VCaP and LNCaP cells (Supplementary Figure S1). Therefore, salinomycin reduces the growth of prostate cancer cells, but does not induce apoptosis.

### Salinomycin inhibits ALDH activity and reduces CD44 cell fraction

**Figure 1:**
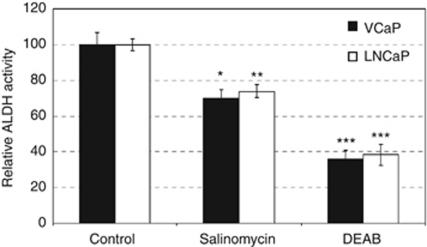
Salinomycin inhibits aldehyde dehydrogenase (ALDH) activity in prostate cancer cells. ALDH activity was measured with Aldefluor assay in response to 1-mmol l^−1^ exposures of salinomycin or control for 48 h in VCaP and LNCaP cells. ALDH inhibitor diethylaminobenzaldehyde (DEAB) was used as negative control. Asterisks indicate statistical significance. ^*^P<0.05; ^**^P<0.01; ^***^P<0.001.

**Figure 2:**
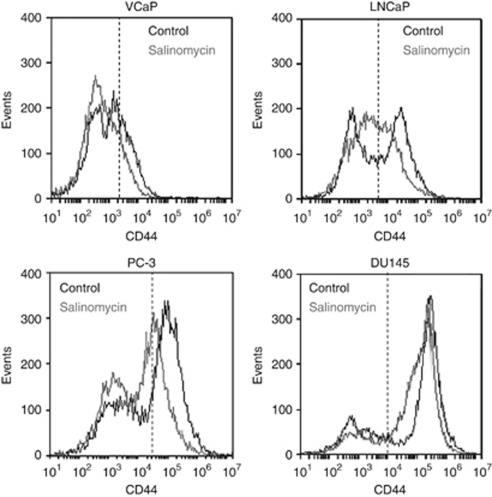
Salinomycin reduces the fraction of CD44^+^ cells in prostate cancer cells. VCaP, LNCaP, PC-3 and DU-145 cells were stained with CD44 antibody and the fluorescence intensities were identified for cells exposed to 6 h of salinomycin or DMSO control.

Aldehyde dehydrogenase activity is considered as a marker for stem cell potential (Moreb, 2008) and tumourigenic prostate cancer cells (Burger *et al*, 2009; Li *et al*, 2010; Yu *et al*, 2011). As salinomycin has been previously identified as a cancer stem cell growth inhibitor (Gupta *et al*, 2009), we studied whether salinomycin reduces ALDH activity in VCaP and LNCaP prostate cancer cells. The results indicated that salinomycin exposure for 48 h resulted in a significant decrease in ALDH activity in VCaP (by 30%) and LNCaP (by 26%) cells (Figure 1). To confirm the impact on prostate cancer stem cells, the intensity of cancer stem cell marker CD44 in VCaP, LNCaP, PC-3 and DU-145 prostate cancer cells was studied in response salinomycin exposure (1 *μ*M) using fluorescence-activated cell-sorting analysis. The results indicated that salinomycin reduced the amount of CD44^+^ cells in all prostate cancer cells tested already after 6-h exposure (Figure 2). The division of CD44-positive and -negative cells (shown as scattered lines in Figure 2) in all cell lines are presented in Supplementary Figure S2. Taken together, these results indicate that salinomycin decreases ALDH activity and CD44^+^ cancer stem cell fraction in cultured prostate cancer cells.

### Salinomycin reduces AR signalling and alters genes involved in lipid metabolism, cell-cycle checkpoint regulation and oxidative stress response

**Figure 3:**
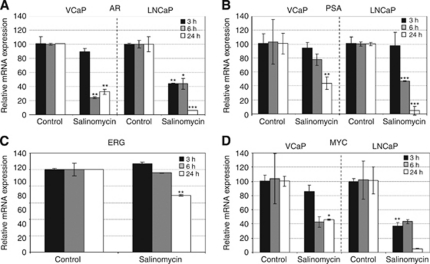
Salinomycin reduces the expression levels of key prostate cancer oncogenes, androgen receptor (AR), ERG and MYC in prostate cancer cells. (**A**) AR, (**B**) PSA, (**C**) ERG and (**D**) MYC mRNA expression in response to 3-, 6-, and 24-h salinomycin exposure in VCaP and LNCaP cells. Asterisks indicate statistical significance. ^*^P<0.05; ^**^P<0.01; ^***^P<0.001.

To get additional insights into the salinomycin-induced alterations in prostate cancer cells, quantitative RT-PCR and genome-wide gene expression profiles were analysed in prostate cancer cells. First, the effect of salinomycin on the expression of key genes involved in prostate cancer, AR, ERG and MYC was studied. The results indicated that salinomycin decreased AR mRNA levels in VCaP and in LNCaP cells (by 32% and 56% at 6-h time points; Figure 3A). Accordingly, a decrease in the levels of PSA mRNA, considered as a marker of the activity of androgen signalling, was also seen in response to salinomycin exposure for 24 h in VCaP and in LNCaP cells (by 50 and 90% Figure 3B). The decrease in AR and PSA protein levels was also confirmed (Supplementary Figure S3). In addition, salinomycin reduced ERG mRNA expression in VCaP cells (by 30% Figure 3C) and MYC mRNA levels in both VCaP and LNCaP cells (by 60% Figure 3D).

Second, salinomycin-induced alterations in gene expression profiling were analysed in VCaP cells using IPA. The most prominent changes at all time points studied were seen in lipid metabolism and in cholesterol and steroid biosynthesis (Supplementary Tables S2 and S3). In addition, cell-cycle checkpoint regulation and aryl hydrocarbon receptor (AhR) signalling were deregulated at all time points, with the most significant changes seen after a 24-h exposure. However, the level of AhR mRNA were not changed (Supplementary Figure S4). Moreover, changes were seen in oxidative stress response, mitochondrial membrane characteristics and cellular movement (Supplementary Tables S2 and S3). Taken together, these results indicate that salinomycin reduces the expression of key prostate cancer oncogenes and AR signalling as well as causes alterations in steroid biosynthesis, cell-cycle checkpoint regulation, AhR signalling and oxidative stress response in prostate cancer cells.

### Salinomycin induces oxidative stress

**Figure 4:**
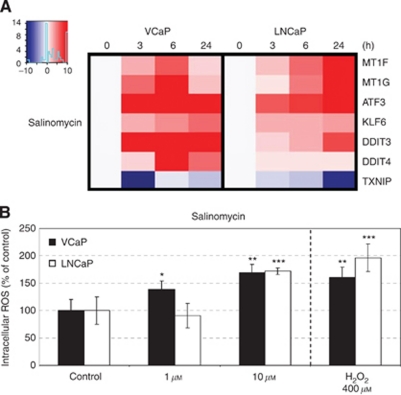
(**A**) Salinomycin induces the gene expression signature characteristic of oxidative stress induction in prostate cancer cells. The expressions of MT1F, MT1G, ATF3, KLF6, DDIT3, DDIT4 and TXNIP mRNAs in VCaP and LNCaP cells in response to salinomycin exposure for 3, 6 and 24 h. (**B**) Salinomycin induces the level of intracellular reactive oxygen species in prostate cancer cells. Reactive oxygen species generation in response to salinomycin exposure in VCaP and LNCaP cells detected with carboxy-H2DCFDA. Hydrogen peroxide (400 mmol l^−1^) exposure for 4 h was used as a positive control. Asterisks indicate statistical significance. ^*^P<0.05; ^**^P<0.01; ^***^P<0.001.

The alterations in oxidative stress response were seen in the gene expression analysis. Thus, the expression of oxidative stress markers TXNIP, MT1G, MT1F, ATF3, KLF6, DDIT3 and DDIT4 were further validated using quantitative real-time PCR. The results indicated that salinomycin induced the gene expression signature characteristic of oxidative stress induction in both VCaP and LNCaP cells (Figure 4A, Supplementary Figure S5 and S6). However, the induction was not as strong in LNCaP cells as in VCaP cells. To validate the oxidative stress induction, the increase in intracellular level of ROS was studied using carboxy-H2DCFDA marker. The results confirmed that salinomycin increases ROS in both VCaP and LNCaP cells (Figure 4B).

### Salinomycin shows similar effects as niclosamide and terfenadine

Analysis of connectivity map data was used to identify compounds with similar or opposite effects as salinomycin. The differentially expressed genes in response to salinomycin exposure for 6 h in VCaP cells were compared with the >7000 expression profiles representing drug responses to >1309 compounds. Niclosamide and terfenadine were the most enriched compounds altering gene expression in the same direction as salinomycin (Supplementary Table S4). Interestingly, niclosamide is an antihelmintic drug that was recently shown to inactivate the NF-*κ*B pathway and to generate ROS in leukaemic stem cells, whereas terfenadine is an antihistamine that induces apoptosis in melanoma cells and causes massive hydrogen peroxide production in cultured cerebellar neurons (Diaz-Trelles *et al*, 2000; Jangi *et al*, 2008; Jin *et al*, 2010). These results give further support to the fact that salinomycin induces oxidative stress in prostate cancer cells.

### Salinomycin reduces NF-*κ*B pathway activity in prostate cancer cells

**Figure 5:**
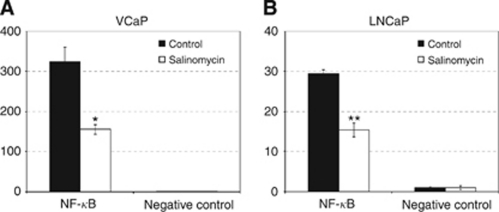
Salinomycin reduces NF-*κ*B activity in prostate cancer cells. The activity of NF-*κ*B and negative control was measured using cancer reporter array in (**A**) VCaP and in (**B**) LNCaP cells in response to 100 nM salinomycin exposure for 24 h and the results were compared with control exposures. Asterisks indicate statistical significance. ^*^P<0.05; ^**^P<0.01.

Studies of the NF-*κ*B inactivator niclosamide suggest that inhibition of NF-*κ*B activity could also have a role in the induction of oxidative stress in salinomycin-exposed prostate cancer cells. Thus, we studied the effects of salinomycin on NF-*κ*B pathway activity using a cancer reporter assay. Interestingly, NF-*κ*B pathway was 10 times higher in VCaP than in LNCaP cells (Figure 5). The results indicated that salinomycin reduces NF-*κ*B activity in VCaP (by 52%) and LNCaP (by 48%) cells (Figure 5). These results support the hypothesis that salinomycin-induced growth inhibition and induction of oxidative stress in prostate cancer cells is mediated by the inhibition of NF-*κ*B activity.

### Salinomycin reduces prostate cancer cell migration

**Figure 6:**
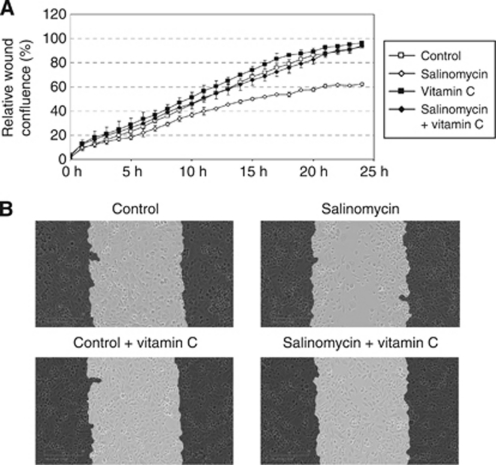
Salinomycin reduces PC-3 prostate cancer cell migration and the effect is antagonised with antioxidant vitamin C. (**A**) Relative wound density in response to salinomycin alone (100 nM, *P*<0.001 for control *vs* salinomycin) and in combination with vitamin C (10 *μ*M) as well as appropriate controls monitored for 24 h. (**B**) The morphological pictures from wound density after 24-h exposures of salinomycin alone and in combination vitamin C. The cells in the beginning of the experiment are marked in black.

The analysis of gene expression profiling results indicated that cellular movement and migration were altered in response to 6-h exposure of salinomycin (Supplementary Table S2). Furthermore, NF-*κ*B and ALDH activities as well as CD44^+^ cells have all been highlighted as markers of prostate cancer cell migration (Klarmann *et al*, 2009; Zhang *et al*, 2009b; van den Hoogen *et al*, 2010). Thus, we explored the effect of salinomycin on prostate cancer cell motility using a wound-healing assay. As VCaP and LNCaP cells do not migrate and therefore are not suitable for this experiment, PC-3 prostate cancer cells were used. Cells were exposed to salinomycin (100 nM) and wound confluence was monitored every hour for 24 h. The results indicated that salinomycin reduces the migration of PC-3 cells. As the EC_50_ value of salinomycin in PC-3 cells in response to 48-h exposure was higher than 1 *μ*M, the salinomycin-induced anti-migratory effect is not due to inhibition of cell proliferation. This was confirmed also by cell viability assay (data not shown). To study whether salinomycin-induced oxidative stress has a role in the migratory phenotype, PC-3 cells were exposed to antioxidant vitamin C (10 *μ*M) and salinomycin (100 nM) and the wound confluence was monitored for 24 h. The results show that vitamin C antagonises the anti-migratory effect of salinomycin in PC-3 cells, indicating that induction of oxidative stress inhibits prostate cancer cell migration (Figure 6).

### Salinomycin induces 7-ketocholesterol and aldosterone levels as well as reduces progesterone and pregnenolone levels

**Figure 7:**
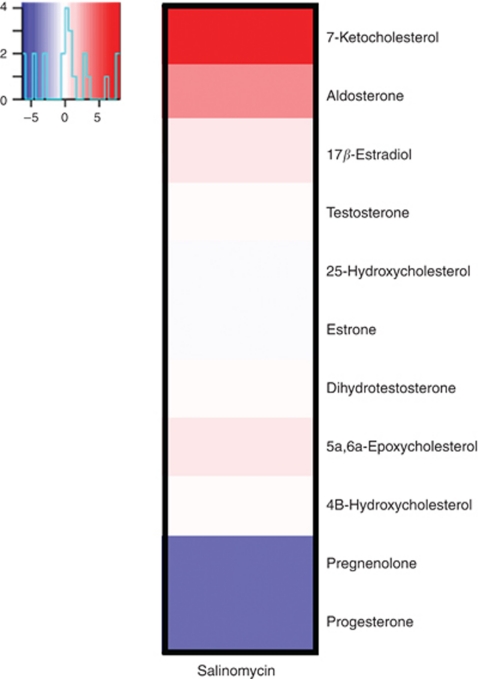
Salinomycin induces the levels of 7-ketocholesterol and aldosterone and reduces the levels of progesterone and pregnenolone. The cells were exposed to salinomycin (1 *μ*M) for 6 h and the steroid profile was measured with gas chromatography-mass spectrometry.

As salinomycin altered lipid metabolism and steroid biosynthesis as well as reduced AR signalling, steroid profiling was performed in salinomycin-exposed VCaP cells using GC-MS. The results showed that the most prominent changes in response to salinomycin exposure were the increase in 7-ketocholesterol and aldosterone levels as well as the decrease in progesterone and pregnenolone (Figure 7, Supplementary Table S5). Interestingly, 7-ketocholesterol is a cholesterol oxidising product that induces ROS, apoptosis, mitochondrial DNA damage and dysfunction as well as ER stress (Leonarduzzi *et al*, 2006; Lee *et al*, 2009; Gramajo *et al*, 2010). Recently, aldosterone has also been linked to oxidative stress induction (Calo *et al*, 2010; Queisser *et al*, 2011). In contrast, progesterone and pregnenolone are precursors of androgens and progesterone has shown to have antioxidant properties (Ozacmak and Sayan, 2009). Moreover, progesterone has recently been identified as an inducer of adult mammary stem cell expansion (Joshi *et al*, 2010). Taken together, salinomycin induces steroids involved in oxidative stress induction and reduces steroids that sustain antioxidative capacity and induce stem cell expansion.

## Discussion

The treatment options for advanced prostate cancer are limited. We have recently identified monensin, a widely used antibiotic in poultry, as an inducer of oxidative stress and a potent inhibitor of prostate cancer cell growth. Antibiotic compound salinomycin shares a similar structure as monensin and was recently identified as a novel cancer stem cell inhibitor in breast cancer (Miyazaki *et al*, 1974; Danforth *et al*, 1977; Mahmoudi *et al*, 2006; Gupta *et al*, 2009). The inhibition of cancer stem cells has been suggested as a potential new therapeutic option for advanced and metastatic cancer (Clayton and Mousa, 2011). In addition, cancer stem cells are known to have strong antioxidative defence mechanisms and the reduction of antioxidative genes has been suggested as a means to target cancer stem cells (Kobayashi and Suda, 2012). Interestingly, many key oncogenes in prostate cancer cells are also known to induce antioxidative properties. However, cancer stem cell inhibitors have not been identified for prostate cancer therapy. Thus, here we studied the growth inhibitory potential and the mechanism of action of salinomycin in cultured human prostate cancer cells.

In this study, we showed for the first time that prostate cancer cells, but not non-tumourigenic prostate epithelial cells, are sensitive to salinomycin. Although salinomycin reduced prostate cancer cell growth, it did not markedly induce caspase 3- and 7-mediated apoptosis. We also showed that salinomycin had an impact on prostate cancer stem cell population using ALDH activity and the amount of CD44^+^ cells as markers. Aldehyde dehydrogenase activity has been used as a marker of prostate cancer stem cells and proposed as a marker of poor outcome in prostate cancer (Davydov *et al*, 2004; Burger *et al*, 2009; Zhang *et al*, 2009a; Li *et al*, 2010; Yu *et al*, 2011). A decrease in cancer stem cell population in response to salinomycin exposure has been shown with breast leukaemia and lung cancer (Gupta *et al*, 2009; Fuchs *et al*, 2009, 2010; Wang, 2011). Aldehyde dehydrogenase enzymes catalyse the dehydrogenation of aldehydes, and thereby protect cells from oxidative stress (Davydov *et al*, 2004; Klarmann *et al*, 2009; Zhang *et al*, 2009a). In addition, CD44 is known to defend cancer cells against oxidative stress by increasing reduced glutathione synthesis (Ishimoto *et al*, 2011). We also identified that salinomycin reduced the expression of prostate cancer oncogenes, *MYC*, *AR* and *ERG,* which are known to have antioxidative properties (Tam *et al*, 2003; Benassi *et al*, 2006; Pinthus *et al*, 2007; Swanson *et al*, 2011). Thus, the results suggest that salinomycin may inhibit prostate cancer stem cells by impairing the redox control. The altered redox regulation in response to salinomycin exposure was confirmed by the induction of intracellular ROS production as well as the gene expression signature characteristic of oxidative stress. Taken together, the antineoplastic effects of salinomycin resulted from decreased cancer stem cell population, reduced expression of key oncogenes and induction of oxidative stress in cultured prostate cancer cells.

Connectivity map analysis indicated that salinomycin has similar effects as terfenadine, known to induce ROS, and niclosamide, an inhibitor of NF-*κ*B. The decrease in NF-*κ*B activity in salinomycin-exposed cells was validated in prostate cancer cells. Nuclear factor-*κ*B is known to regulate cellular antioxidant defence capacity as well as prostate cancer cell viability, tumourigenesis and metastasis (Gloire *et al*, 2006; Sarkar *et al*, 2008; Gluschnaider *et al*, 2010). Nuclear factor-*κ*B pathway is active in prostate stem-like tumour-initiating cells and its inhibition induces apoptosis in prostate cancer stem cells. Therefore, NF-*κ*B is considered as a promising therapeutic target (Birnie *et al*, 2008; Jin *et al*, 2008; Rajasekhar *et al*, 2011). Nuclear factor-*κ*B was recently shown to be activated by specific TMPRSS2–ERG fusion isoforms, which may explain the 10-fold increase in NF-*κ*B activity seen in VCaP cells in comparison to the LNCaP cells (Wang *et al*, 2010). As NF-*κ*B and ALDH activities as well as CD44^+^ cell population have all been previously shown to regulate prostate cancer cell migration (Klarmann *et al*, 2009; Zhang *et al*, 2009b; van den Hoogen *et al*, 2010), the migratory effect of salinomycin was studied in prostate cancer cells. Our results revealed that salinomycin exposure inhibited prostate cancer cell migration and was antagonised by antioxidant vitamin C, indicating that induction of oxidative stress has an important role in mediating the salinomycin-induced anti-migratorial phenotype. Salinomycin was recently also shown to reduce cell invasion in cultured colorectal cancer cells (Dong *et al*, 2011).

Results from steroid profiling showed that salinomycin reduced the levels of progesterone and pregnelonone, both precursors of androgens. Progesterone is known to induce antioxidative capacity and mammary stem cell expansion (Ozacmak and Sayan, 2009; Joshi *et al*, 2010). Moreover, we show that salinomycin induced the levels of 7-ketocholesterol and aldosterone, which are known to act as oxidative stress inducers (Leonarduzzi *et al*, 2006; Lee *et al*, 2009; Calo *et al*, 2010; Gramajo *et al*, 2010; Queisser *et al*, 2011). The cholesterol oxidation product 7-ketocholesterol is a ligand for AhR and may function as an AhR antagonist (Savouret *et al*, 2001). Aryl hydrocarbon receptor pathway has been suggested to have an essential role in detoxification of foreign chemicals and in the protection against oxidative stress by increasing the expression of ALDH proteins (Lindros *et al*, 1998; Nebert *et al*, 2000; Vrzal *et al*, 2004; Kohle and Bock, 2007). Moreover, AhR is overexpressed in prostate cancer and cancer stem cells and it can bind to NF-*κ*B and promote activation of *MYC* (Kim *et al*, 2000; Blum *et al*, 2009; Gluschnaider *et al*, 2010). Our gene expression analysis results showed that AhR target genes were reduced by salinomycin, although the AhR receptor mRNA levels were not changed. However, many processes known to be modulated via AhR were altered in salinomycin-exposed prostate cancer cells.

In conclusion, our results reveal that the ability of salinomycin to inhibit prostate cancer cell growth and cancer stem cell population, without major effects on non-malignant prostate epithelial cells, is due to the induction of oxidative stress and the reduction of antioxidative properties. Thus, salinomycin and its derivatives may provide a novel selective approach for prostate cancer therapy.

## Figures and Tables

**Table 1:**
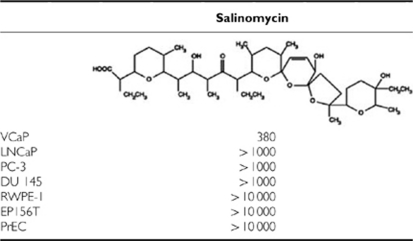
EC_50_ value (nM) for salinomycin in various prostate epithelial cells
