# Supplementary figures and images for: Salinomycin inhibits prostate cancer growth and migration via induction of oxidative stress
Source: Br J Cancer. 2012 Jan 3;106(1):99–106. doi: 10.1038/bjc.2011.530 (PMC3251868; doi:10.1038/bjc.2011.530)

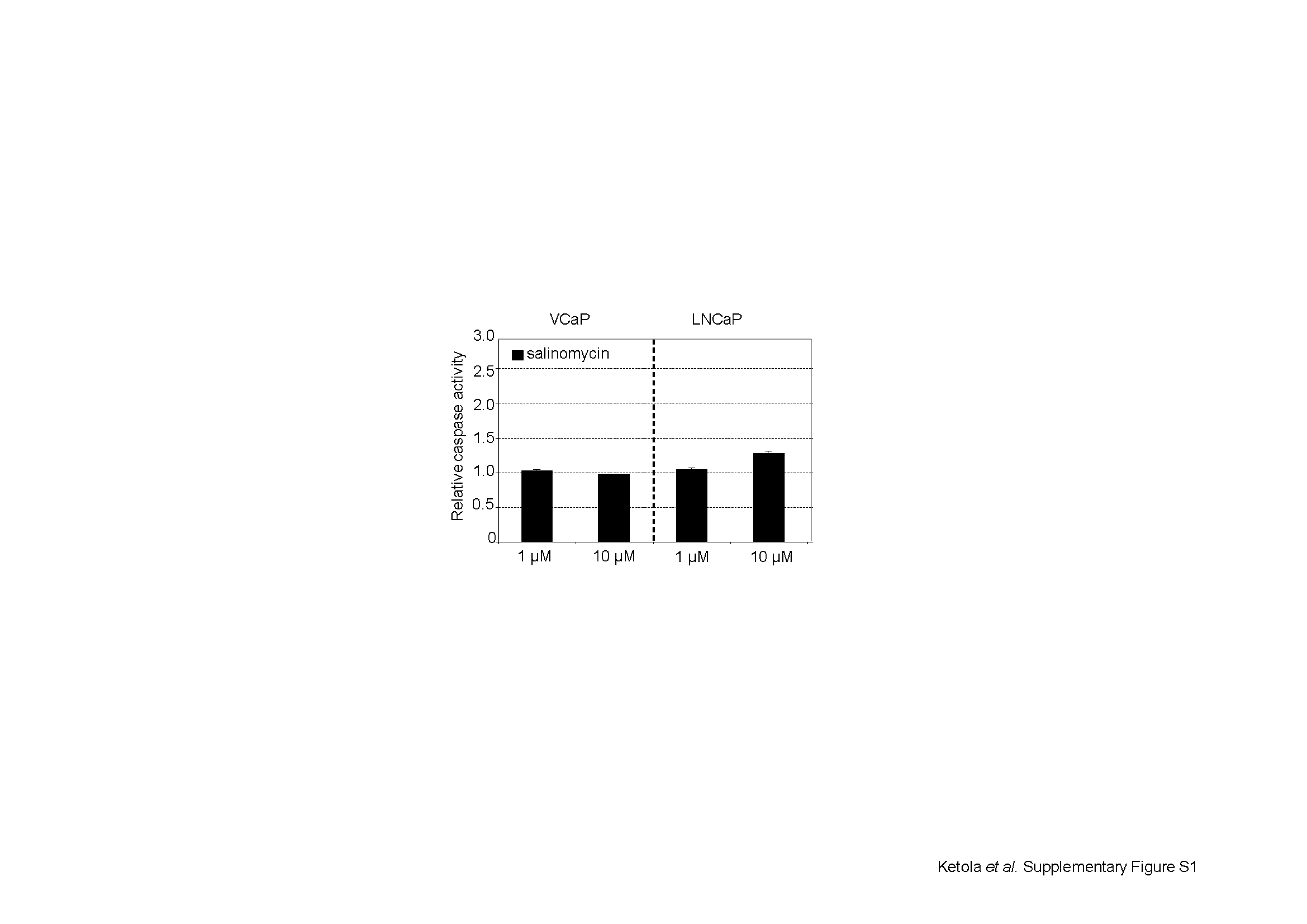

Supplement: Supplementary Figure 1 [file bjc2011530x1.tif]

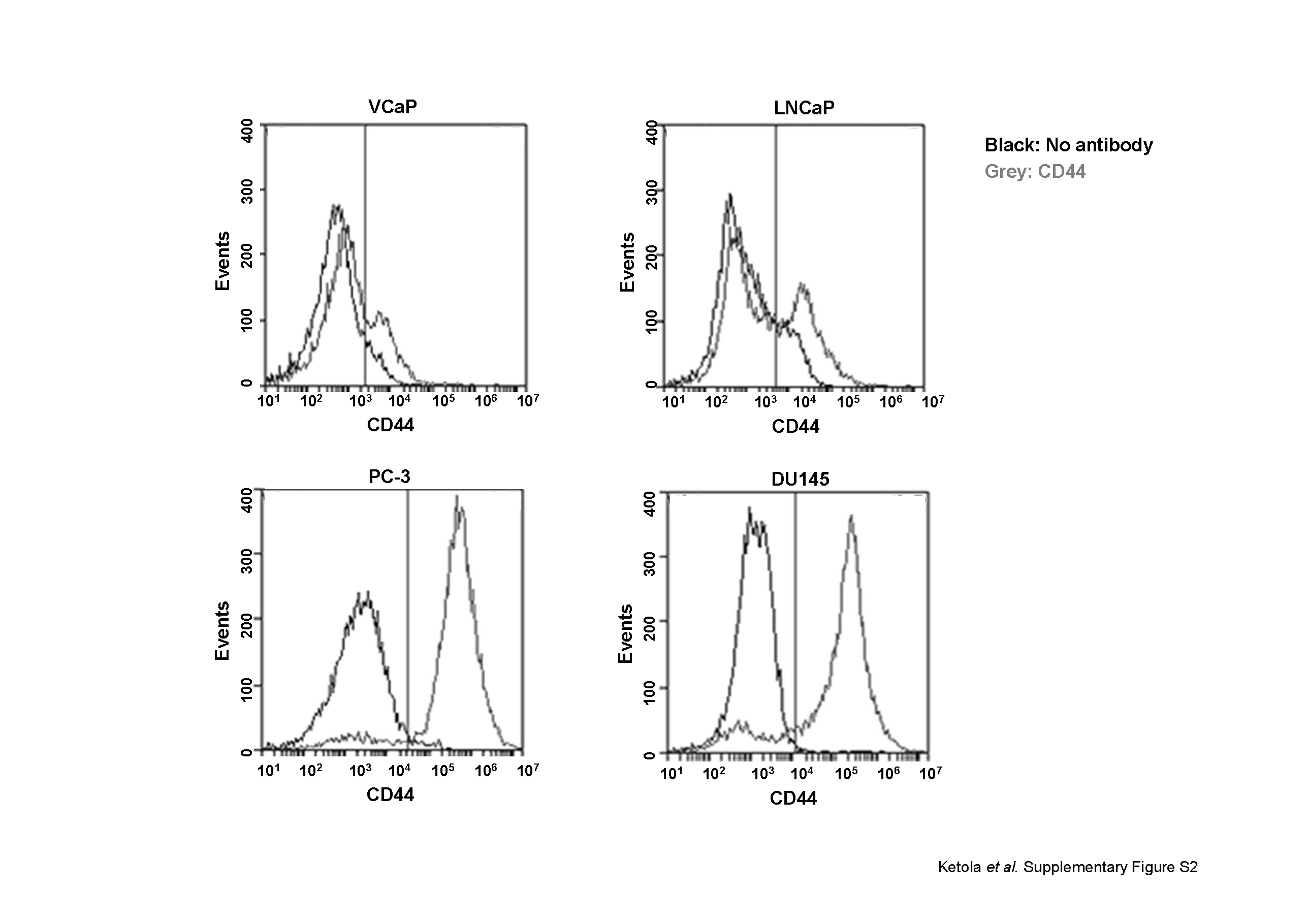

Supplement: Supplementary Figure 2 [file bjc2011530x2.tif]

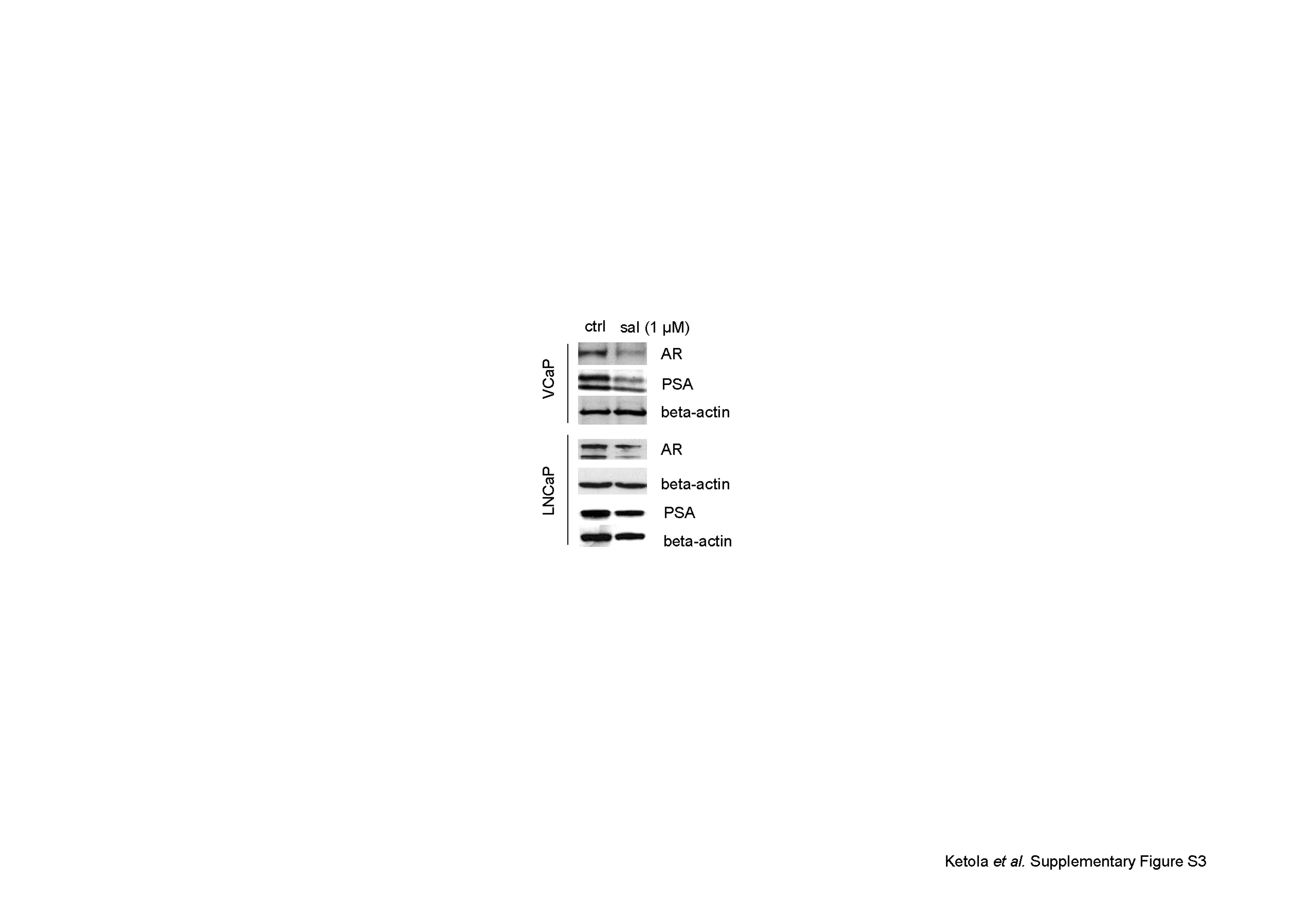

Supplement: Supplementary Figure 3 [file bjc2011530x3.tif]

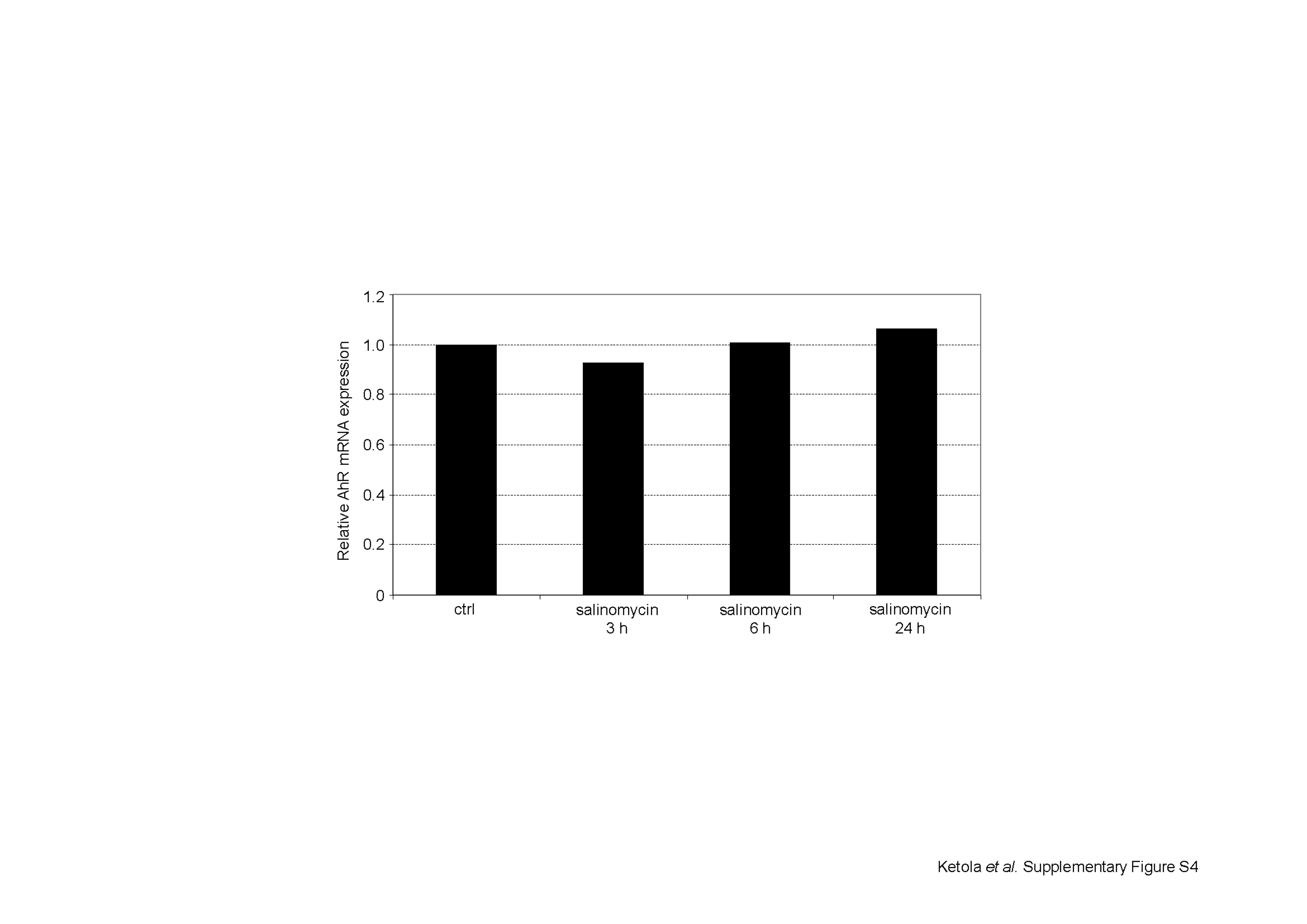

Supplement: Supplementary Figure 4 [file bjc2011530x4.tif]

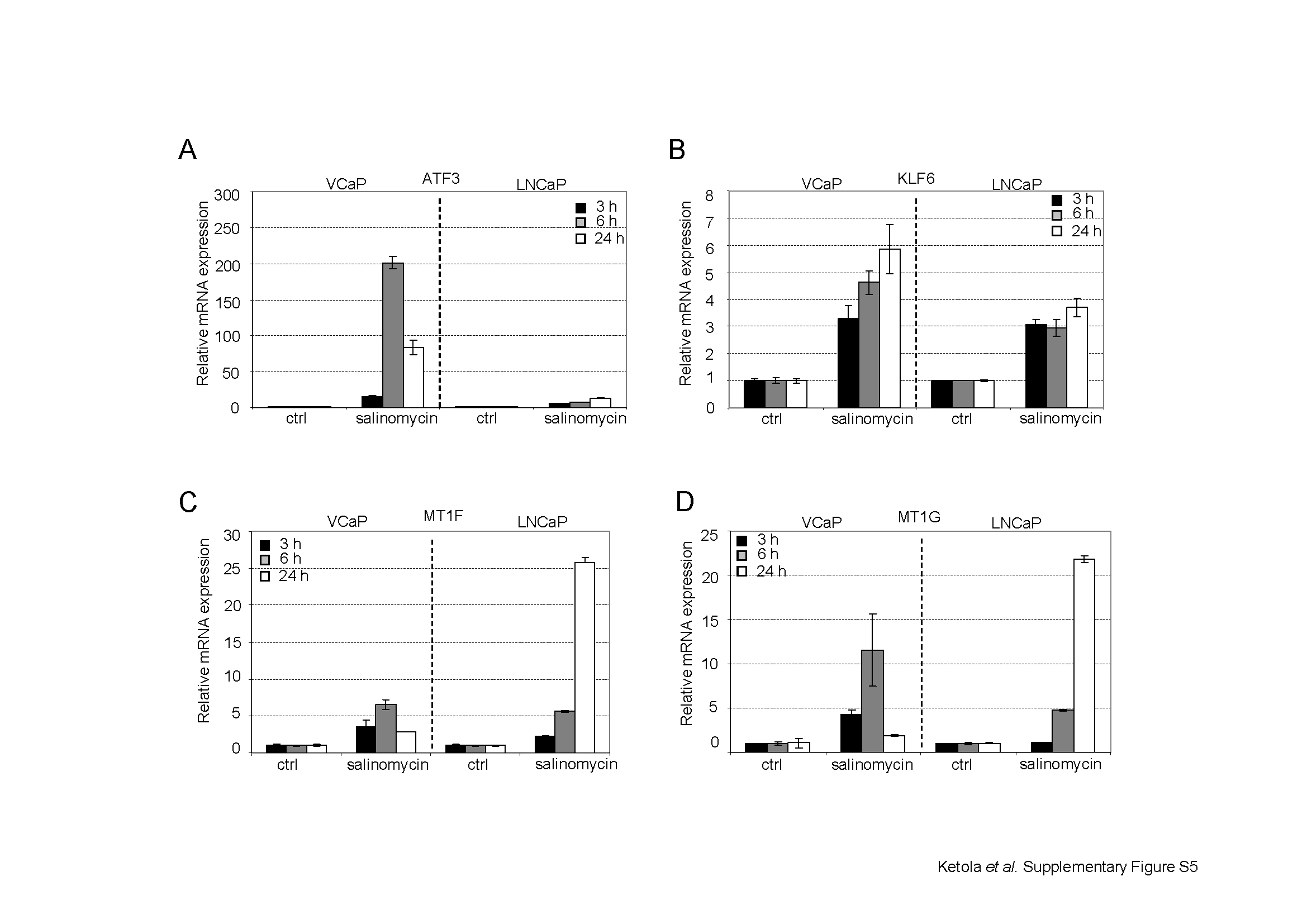

Supplement: Supplementary Figure 5 [file bjc2011530x5.tif]

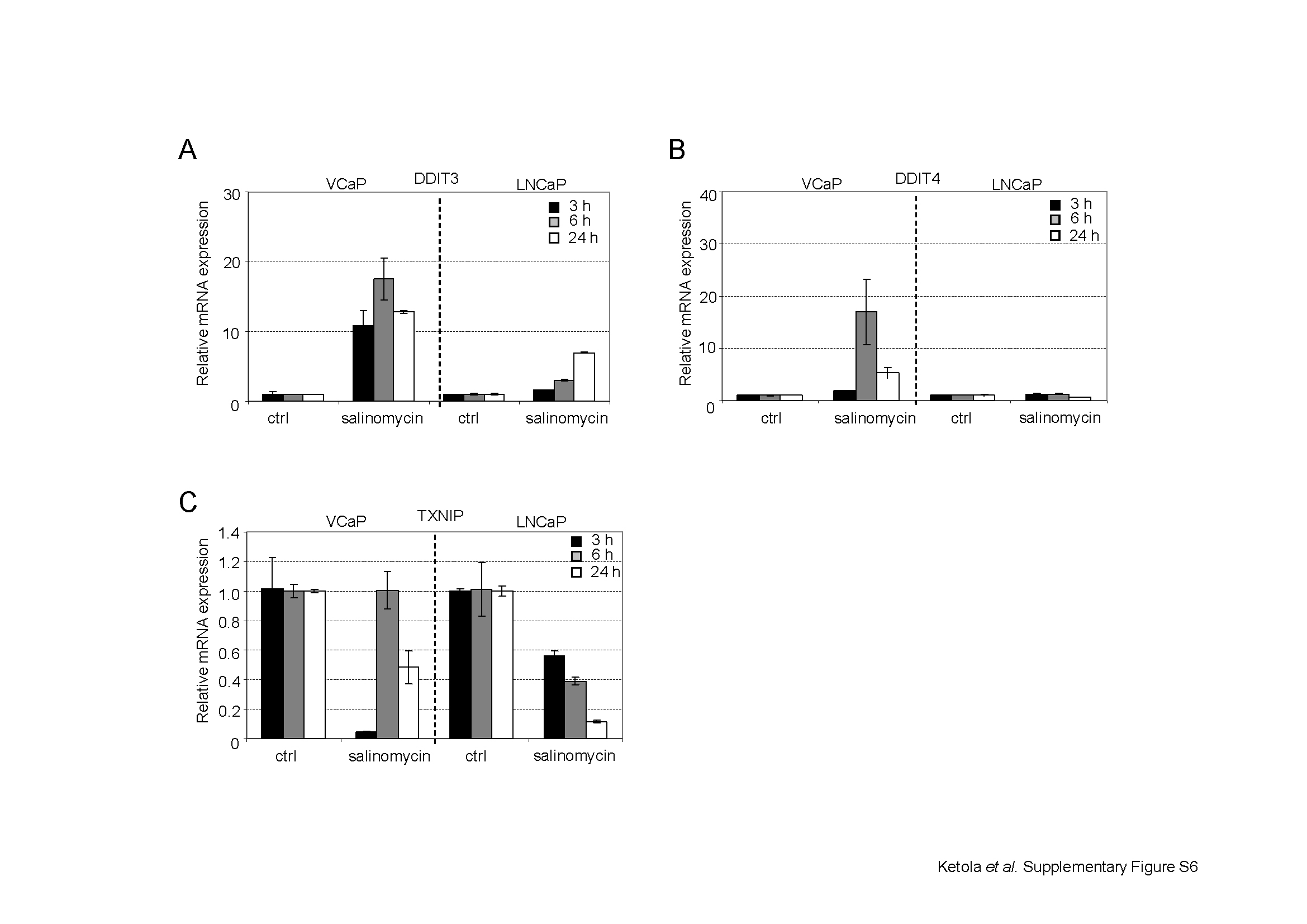

Supplement: Supplementary Figure 6 [file bjc2011530x6.tif]
