# Supplementary material for: Salinomycin inhibits prostate cancer growth and migration via induction of oxidative stress
Source: Br J Cancer. 2012 Jan 3;106(1):99–106. doi: 10.1038/bjc.2011.530 (PMC3251868; doi:10.1038/bjc.2011.530)
Supplement: Supplementary Information [file bjc2011530x12.doc]

# Supplementary files

Supplementary Table S1. Primer sequences of quantitative reverse transcriptase PCRs.

Supplementary Table S2. Gene ontology analysis of differentially expressed genes (fold change > 1.5, p < 0.001) identified with Ingenuity Pathway Analysis (IPA) software in response to 1 µM salinomycin exposure for 3, 6 and 24 hours in VCaP cells.

Supplementary Table S3. Ingenuity toxicity lists of differentially expressed genes (fold change > 1.5, p < 0.001) identified with Ingenuity Pathway Analysis (IPA) software in response to 1 µM salinomycin exposure for 3, 6 and 24 hours in VCaP cells.

Supplementary Table S4. Connectivity map results (enrichment score either > 0.7 or < ‑0.7) with differentially expressed genes (fold change > 0.6, p < 0.01) in response to 6-hour exposure of salinomycin in VCaP cells.

Supplementary Table S5. The levels of steroids 7-ketocholesterol, 17B-estradiol, aldosterone, testosterone, 25-hydroxycholesterol, estrone, dihydrotestosterone, 5a,6a-epoxycholesterol (Mono-TMS), 4B-hydroxycholesterol, pregnenolone and progesterone in response to salinomycin (1 µM) and control exposures for 6 hours.

Supplementary Figure S1. Induction of apoptosis detected by measuring caspase 3 and 7 activations in response to salinomycin exposure (1 µM or 10 µM) for 48 hours in VCaP and LNCaP cells. Data are presented as mean +/- SD from six independent experiments. Stars indicate the significance of the apoptotic induction (*** p<0.001).

Supplementary Figure S2. The intensities of untreated control cells stained with CD44 antibody in VCaP, LNCaP, PC-3 and DU-145 cells (black: no antibody, red: CD44).

Supplementary Figure S3. AR, PSA and β-actin protein expressions in response to salinomycin exposure for 24 hours in VCaP and LNCaP cells.

Supplementary Figure S4. Relative mRNA expression level of aryl hydrocarbon receptor (AhR) analyzed with Illumina in response to salinomycin treatment for 3, 6 and 24 hours in VCaP cells.

Supplementary Figure S5. Expression levels of oxidative stress responsive genes MT1F, MT1G, ATF3 and KLF6 in VCaP and LNCaP cells in response to salinomycin treatment for 3, 6 and 24 hours.

Supplementary Figure S6. Expression levels of oxidative stress responsive genes DDIT3, DDIT4 and TXNIP in VCaP and LNCaP cells in response to salinomycin treatment for 3, 6 and 24 hours.
